# Effects of Mobile-Based Financial Incentive Interventions for Adults at Risk of Developing Hypertension: Feasibility Randomized Controlled Trial

**DOI:** 10.2196/36562

**Published:** 2023-03-24

**Authors:** Amanda Willms, Ryan E Rhodes, Sam Liu

**Affiliations:** 1 School of Exercise Science, Physical and Health Education University of Victoria Victoria, BC Canada; 2 Department of Psychology University of Victoria Victoria, BC Canada

**Keywords:** mHealth, physical activity, financial incentive, hypertension, mobile health, exercise, lifestyle health, cardiovascular disease, mortality, heart disease, incentive, motivation

## Abstract

**Background:**

Hypertension is the leading modifiable risk factor for cardiovascular disease and mortality. Adopting lifestyle modifications, like increasing physical activity (PA), can be an effective strategy in blood pressure (BP) control, but many adults do not meet the PA guidelines. Financial incentive interventions have the power to increase PA levels but are often limited due to cost. Further, mobile health technologies can make these programs more scalable. There is a gap in the literature about the most feasible and effective financial incentive PA framework; thus, pay-per-minute (PPM) and self-funded investment incentive (SFII) frameworks were explored.

**Objective:**

The aims were to (1) determine the feasibility (recruitment, engagement, and acceptability) of an 8-week mobile-based PPM and SFII hypertension prevention PA program and (2) explore the effects of PPM and SFII interventions relative to a control on the PA levels, BP, and PA motivation.

**Methods:**

In total, 55 adults aged 40-65 years not meeting the Canadian PA guidelines were recruited from Facebook and randomized into the following groups: financial incentive groups, PPM or SFII, receiving up to CAD $20 each (at the time of writing: CAD $1=US $0.74), or a control group without financial incentive. PPM participants received CAD $0.02 for each minute of moderate-to-vigorous PA (MVPA) per week up to the PA guidelines and the SFII received CAD $2.50 for each week they met the PA guidelines. Feasibility outcome measures (recruitment, engagement, and acceptability) were assessed. Secondary outcomes included changes in PA outcomes (MVPA and daily steps) relative to baseline were compared among PPM, SFII, and control groups at 4 and 8 weeks using linear regressions. Changes in BP and relative autonomy index relative to baseline were compared among the groups at follow-up.

**Results:**

Participants were randomized to the PPM (n=19), SFII (n=18), or control (n=18) groups. The recruitment, retention rate, and engagement were 77%, 75%, and 65%, respectively. The intervention received overall positive feedback, with 90% of comments praising the intervention structure, financial incentive, and educational materials. Relative to the control at 4 weeks, the PPM and SFII arms increased their MVPA with medium effect (PPM vs control: η^2^_p_=0.06, mean 117.8, SD 514 minutes; SFII vs control: η^2^_p_=0.08, mean 145.3, SD 616 minutes). At 8 weeks, PPM maintained a small effect in MVPA relative to the control (η^2^_p_=0.01, mean 22.8, SD 249 minutes) and SFII displayed a medium effect size (η^2^_p_=0.07, mean 113.8, SD 256 minutes). Small effects were observed for PPM and SFII relative to the control for systolic blood pressure (SBP) and diastolic blood pressure (DBP) (PPM: η^2^_p_=0.12, Δmean SBP 7.1, SD 23.61 mm Hg; η^2^_p_=0.04, Δmean DBP 3.5, SD 6.2 mm Hg; SFII: η^2^_p_=0.01, Δmean SBP −0.4, SD 1.4 mm Hg; η^2^_p_=0.02, Δmean DBP −2.3, SD 7.7 mm Hg) and relative autonomy index (PPM: η^2^_p_=0.01; SFII: η^2^_p_=0.03).

**Conclusions:**

The feasibility metrics and preliminary findings suggest that a future full-scale randomized controlled trial examining the efficacy of PPM and SFII relative to a control is feasible, and studies with longer duration are warranted.

## Introduction

### Background

Hypertension and prehypertension are leading risk factors for strokes, ischemic heart disease, and other vascular diseases, and currently lead to 8.5 million deaths globally [1,2]. Regular physical activity (PA) is a key lifestyle factor for lowering resting blood pressure (BP) and risk for cardiovascular disease [3]. However, PA levels for adults remain low, with more than 1.4 billion adults worldwide being insufficiently active (<150 minutes of moderate-to-vigorous PA [MVPA] per week) [4]. Canadian adults, specifically, 84% of those aged 18-64 years, are not meeting the Canadian PA guidelines of 150 MVPA minutes per week [5]. In-person PA programs have the potential to lower BP but are limited due to accessibility [6], scalability [7], and cost. Mobile health (mHealth) PA interventions have the potential to overcome these barriers [8]; however, they can suffer from poor engagement [9] and behavior adherence [10,11]. Financial incentives, a form of extrinsic reward, have been gaining popularity to be used with PA interventions over the past decade, as they have been shown to effectively increase PA adherence [12], as well as engagement in the program (ie, completing lessons in an education program) [9]. Financial incentives represent a component of behavioral economics, where individuals are rewarded immediately for their actions to reduce what is called a present bias [13]. Researchers have explored different incentive interventions, manipulating goal setting, financial incentive amount, delivery, and timing. Currently, consensus has not yet been determined for the most effective financial incentive intervention for the prevention of hypertension [14].

A recent systematic review reported that both gain and loss-framed financial incentives can promote PA outcomes (leisure-time PA, walking behavior, PA guidelines, kilocalories expended, and total PA) with small-to-moderate effect [15]. Carrot Rewards (Carrot Insights Inc.), a mHealth app that rewarded individuals’ daily rewards (CAD $0.04 per day; at the time of writing: CAD $1=US $0.74) in the form of loyalty rewards for reaching their step goals, received attention for its success in Canada [16]. This structure of financial incentive is known as pay-for-performance and has been gaining popularity in recent years due to its success [17]. However, on a population level, even modest incentives for PA may not be feasible or sustainable long-term due to cost [18]. Thus, a more sustainable financial incentive model is needed for PA promotion.

An innovative and sustainable solution could be a self-funded investment incentive (SFII). This funding model is similar to a social impact bond, a contract between a governing authority and the public sector to produce better social outcomes, that is, better health [19]. The SFII incorporates tactics from the social impact bond structure by rewarding participants in a pay-for-success model, where once the goals agreed upon are met, financial and social (ie, health) benefits are made. Currently, the effectiveness of the SFII and pay-per-minute (PPM) has not been previously evaluated. Thus, a feasibility study is needed to explore the preliminary efficacy of these financial incentive interventions.

### Objectives

The primary objective of this study was to determine the feasibility (recruitment, engagement, and acceptability) of an 8-week mobile-based PPM and SFII hypertension prevention program. The secondary objectives of this study were to explore the effects of PPM and SFII interventions relative to a control on PA levels, BP, and PA motivation following the intervention.

### Hypotheses

Based on previous literature, it was hypothesized that >70% of interested individuals would be recruited [20,21], engagement rates would be >60% [22], and >80% of participants would find the study acceptable [21]. For the secondary objectives, it was hypothesized that those in the PPM or SFII arms, relative to the control, would show a small-to-moderate effect size in improving MVPA and daily steps at 4 and 8 weeks [15]. Further, those who were receiving a financial incentive would display an increase in relative autonomy and have a small-to-moderate effect on the improvements of resting BP at follow-up, relative to the control group.

## Methods

### Study Design

This randomized feasibility pilot study aligns with the goals of phase IIb of the ORBIT model to determine the feasibility of conducting a trial of a full intervention [23]. This 8-week feasibility trial was conducted between April and August 2021, and the participants were recruited through Facebook ads. Simple randomization was used to assign the participants to 1 of the 2 financial incentive intervention groups (PPM or SFII) or a control group.

### Ethics Approval

All participants provided consent before the start of the study. Ethics approval for this study was obtained through the Human Research Ethics Board at the University of Victoria (protocol 20-0016). All participants provided written informed consent and were informed that their details would be deidentifiable through a unique participant ID and anonymous email address for accessing study content. Independent of the study group, all participants received CAD $20.

### Study Participants

Adults living in British Columbia, Canada, were recruited through Facebook. To be eligible for this study, participants needed to be (1) 40-65 years old, (2) not meeting the Canadian PA guidelines of 150 minutes of MVPA per week (assessed by the Get Active Questionnaire [24]), (3) were fluent in English, and (4) have normal to corrected-normal vision. Participants were excluded if they had a diagnosis of diabetes, other heart conditions, or other mobility restrictions.

### Study Groups

#### PPM Financial Incentive Group

Participants were introduced to an 8-week Healthy Hearts education program, with 1 lesson during the baseline week and 3 lessons per week throughout the 8-week intervention (25 lessons in total). Healthy Hearts aimed to build exercise intention by highlighting the benefits of PA and encouraging goal-setting and self-monitoring. The program was built based on the Multi-Process Action Control (M-PAC) framework. M-PAC addresses the intention-behavior gap through the understanding that ongoing reflective processes (ie, affective attitude and perceived opportunity) and regulation processes (behavioral and cognitive tactics to maintain intention focus) are necessary for one’s intention to become active and that the maintenance of behavior is supported by habit and identity, which can be categorized as reflexive processes [22-25]. Following this framework, the lessons started with intention formation (lessons 1-10), then moved into action control adoption (lessons 11-19), and concluded with action control maintenance (lessons 20-25). Numerous behavior change techniques [25] were used throughout the lessons to support the participant’s advancement through the M-PAC constructs and develop positive exercise habits and exercise identity. Financial incentives are also an effective behavior change technique that can improve engagement in PA interventions [26,27].

Similar to previous “pay-per-minute” studies [28], participants in this intervention arm were rewarded CAD $0.02 for each minute of MVPA tracked through the Fitbit (Fitbit Inc). The maximum amount of money that can be earned per week was CAD $2.50 and CAD $20 for all 8 weeks, which would be rewarded if the individual meets or exceeds 150 minutes of MVPA, the Canadian PA guidelines for adults [29]. Participants were emailed each week informing them which lessons to complete and were notified that they can ask for their current earnings in the study. Participants were compensated after they completed the intervention. If the participant dropped out of the intervention, they were compensated for the number of weeks that they have completed.

#### SFII Financial Incentive Group

**Figure 1:**
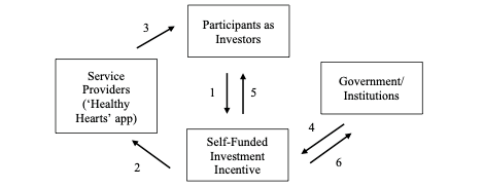
Self-funded investment incentive.

As previously mentioned, the SFII employed in this had similarities to the structure of a social impact bond. It differs in that the participants in the hypertension prevention program acted as both investors and as recipients of the program (eg, the hypertension prevention program). Further, this incentive program encouraged adherence to a PA program by having participants commit a mock investment through a contract. The SFII for this study follows a 6-step structure, broken down into the following (Figure 1). In step 1, it was recognized that participants who received the intervention were the investors. Similar to the traditional social impact bond, participants invested money into the SFII. In steps 2 and 3, the SFII funds were used as a reward in the intervention. Steps 4 and 5 recognize that if the participant reached the desired PA outcome, then the government or private institution would pay the participants for reaching their goal. A unique feature of the SFII is highlighted in step 6. That is, the money invested into the SFII may be reinvested by the government or private institutions. The interest gained from the investment by the government can then be used to pay for the desired outcome in steps 4 and 5. Based on the S&*P* 500, stock index funds over the last 30 years have shown an average investment return between 5% and 8% per year [30]. Thus, in the SFII, a 5% rate of return on investment was used for participants if they achieved the Canadian PA guidelines.

Participants were given the same education program as the PPM group. However, the financial incentive differed. Participants in this intervention arm signed a mock contract committing to invest CAD $400 into their health for the duration of the 8-week program. No money was taken from the participants; however, they were encouraged to put this money aside for the duration of the study. Participants received a percentage of return on this initial investment based on the number of weeks they successfully met the Canadian PA guidelines, as recorded by their Fitbit. If a participant in this group met the goal for 0-2 weeks of the intervention, they received a 0% return. If a participant met the goals for 3-4 weeks of the intervention, they received a 1.5% return on this investment, which is equivalent to CAD $6. If a participant met their goal for 5-7 weeks of the intervention, they received a 3% return on investment or CAD $12. Lastly, the maximum return on investment is 5% or CAD $20, and this was rewarded if the participant met the goal for all 8 weeks of the intervention. A 5% investment return was chosen based on the annualized S&*P* 500 stock based on the last 50 years [30]. Similar to the PPM group, participants were emailed each week informing them which lessons to complete and were told they can ask for their current earnings in the study. Participants were compensated after they completed the intervention. If the participant dropped out of the intervention, they were compensated for the number of weeks they completed.

#### Control Group

To match the weekly intervention delivery frequency, participants received 1 email per week with contents from the web-based source HealthLinkBC [31], Canadian HT Education Program [32], and Health Seekers through the Heart and Stroke Foundation [33]. The information delivered to participants in the control arm included PA and heart health benefits and general PA tools and logs.

### Primary Outcome Measures

#### Recruitment

The recruitment rate was calculated by dividing the number of individuals who enrolled in the study by the number of individuals who were eligible to enroll. This value was then divided by the number of months of recruitment [34]. Screening to enrollment ratio was calculated by dividing the number of individuals who attended the eligibility meeting by the number of participants enrolled in the study [35]. Retention was calculated by dividing the number of participants who completed the study by the number of participants who enrolled in the study [23].

#### App Engagement

Lesson completion data were downloaded from the Pathverse Admin portal (Pathverse Inc). Engagement was defined by the number of lessons that the intervention groups completed through the Pathverse app. There are a total of 25 lessons in the program.

#### Acceptability

Acceptability was measured postintervention through virtual semistructured interviews between the participant and the researcher. Thematic analysis was conducted to analyze and report themes from the semistructured interviews [36], and responses were divided into positive feedback and recommendations for improvement.

### Secondary Outcome Measures

#### Physical Activity Levels

Fitbit devices [37] are a validated tool to measure MVPA over a 7-day period [38]. Activity data from the Fitbit website were downloaded, and activity categorized as “minutes fairly active” and “minutes very active” were summed to accumulate the moderate and vigorous PA minutes, respectively [39].

Step data were downloaded throughout the intervention from the individual’s Fitbit account. The validity and reliability of using Fitbit to measure daily steps have been previously been established [39]. An extremely high or low step count (ie, 2 SD from the population mean) were identified throughout the week to ensure that the participants wore the Fitbit for the entire day. Average daily step count was calculated using 3 randomly selected days during the week and 1 day from the weekend, which is in keeping with conventional procedures for estimating daily step count [40].

#### Blood Pressure

Self-report BP was collected. Participants were emailed instructions on how to measure their BP, per HT Canada to self-report an average of their 3 most recent BP measurements [32]. These instructions included no coffee or smoking 30 minutes prior, rest quietly before measuring, keep feet flat on the floor, and to place a BP cuff on a bare left arm.

#### Physical Activity Motivation

The Behavioral Regulation in Exercise Questionnaire (BREQ-3) [41,42] was used to measure PA motivation. The items on the BREQ-3 include amotivation (Cronbach α=.83), external (Cronbach α=.79), introjected (Cronbach α=.80), identified (Cronbach α=.73), integrated (Cronbach α=.87) [43], and intrinsic (Cronbach α=.86) regulation. The relative autonomy index (RAI) was calculated using the BREQ-3 questionnaire with the following formula: RAI = (amotivation × (−3)) + (external regulation × (−2)) + (introjected regulation × (−1)) + (identified regulation) + (integrated regulation × 2) + (intrinsic regulation × 3). Scores on the RAI range from +20 to −24, with higher scores indicating more autonomous motivation and lower scores indicating more extrinsic motivation [44].

### Procedure

The entire study was conducted digitally due to the COVID-19 pandemic. Interested individuals responded to a Facebook ad and were then contacted to arrange an initial web-based eligibility meeting. This first eligibility meeting was no longer than 30 minutes. Once the consent form was signed and returned, a baseline meeting was scheduled, and a Fitbit Inspire 2 [37] was mailed to the participant.

At the virtual baseline meeting, participants completed the baseline questionnaire (demographic information and the BREQ-3 [41,42]) and reported their most recent resting BP measurement. Anonymous login credentials were given for each participant to log in to a Fitbit account. Before starting the intervention, participants were asked to complete 1 week of baseline testing to collect MVPA and steps using their Fitbit. MVPA and steps were also recorded at 4 and 8 weeks. Participants in the PPM and SFII groups accessed the intervention by downloading the Pathverse app [45] on their smartphone. Pathverse is a no-code app development platform that enables researchers to deliver relevant mHealth content to consented participants. Following the 8-week intervention, participants were asked to complete the same study questionnaire (BREQ-3) as baseline and report their most recent resting BP readings. Participants also completed a semistructured interview to record program acceptability and feedback. All amounts were recorded in Canadian dollars and a currency exchange rate of CAD $1=US $0.78 is applicable at the time of publication.

### Statistical Analysis

#### Primary Outcomes

Descriptive statistics were used to determine the feasibility (recruitment, engagement, and acceptability) of an 8-week mobile-based PPM and SFII hypertension prevention program. The follow-up semistructured interviews were analyzed using thematic analysis (ie, overall positive and negative comments and future program changes) [36].

#### Secondary Outcomes

The changes in MVPA, daily steps, SBP, DBP, and RAI were analyzed using an intention-to-treat approach [46]. Baseline measures were carried forward to deal with missing follow-up data [47]. Delta values for MVPA and steps were calculated with data from baseline, 4 weeks, and 8 weeks. Multiple linear regression was used to evaluate whether changes from baseline to 4 weeks and from baseline to 8 weeks for PA were significantly different between PPM, SFII, and control. Each linear regression model was adjusted for the baseline value of each dependent variable. Similarly, analysis of BP and RAI was calculated with data from baseline and follow-up, and each linear regression model was adjusted for baseline value of each dependent variable. Partial eta squared (η^2^_p_) effect size values were calculated to determine intervention effects. An η^2^_p_=0.01 indicated a small effect, η^2^_p_=0.06 indicated a medium effect and η^2^_p_=0.14 indicated a large effect [48]. Due to the sample size in this feasibility study, partial eta-squared effect sizes were used to provide preliminary evidence that the outcomes are as hypothesized.

## Results

### Participants

**Figure 2:**
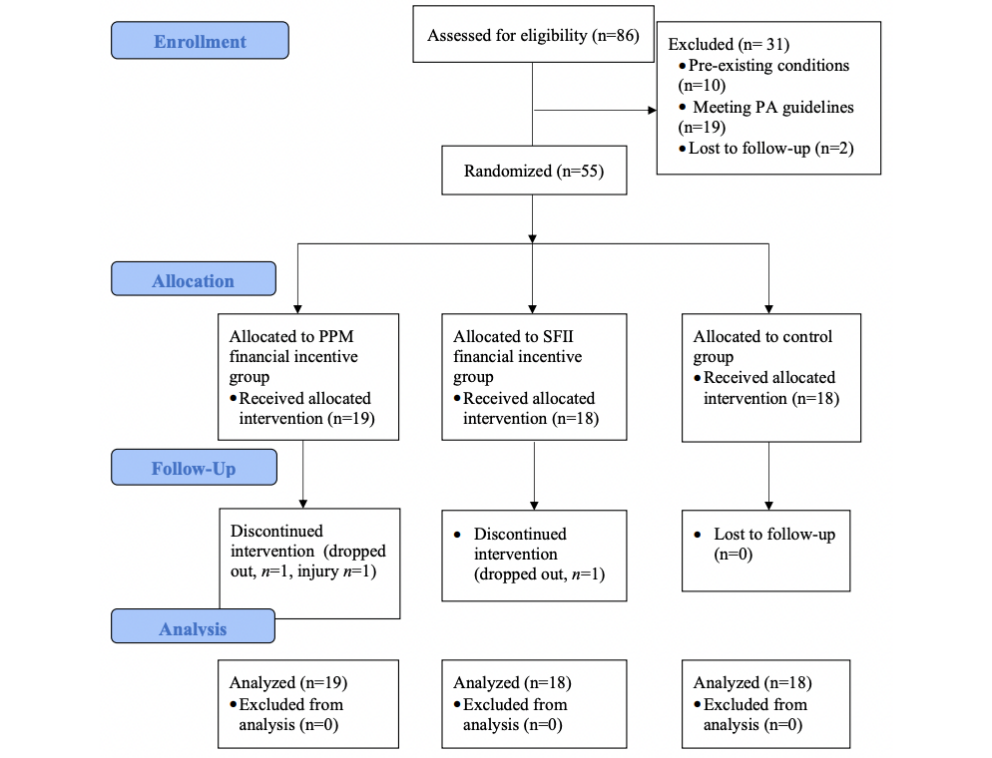
CONSORT (Consolidated Standards of Reporting Trials) flow diagram of enrolment, allocation, follow-up, and analysis. PA: physical activity; PPM: pay-per-minute; SFII: self-funded investment incentive.

Consolidated standards of reporting trials flow diagram outlining participant recruitment is shown in Figure 2. A total of 86 individuals replied to an initial screening email after expressing interest to participate in the study through Facebook during the 5-week recruitment period. In total, 31 participants were excluded because they did not meet the inclusion criteria (n=29) and did not continue to respond to emails (n=2). These participants were then allocated to either the PPM financial incentive group (n=18), the SFII financial incentive group (n=18), or the control group (n=18).

Two participants in the PPM financial incentive group discontinued the intervention, 1 dropped out and stopped syncing their Fitbit, and 1 dropped out due to an injury not related to the study. One participant in the SFII group dropped out and stopped syncing their Fitbit. All participants (n=18, 100%) allocated to the control arm successfully completed the intervention and follow-up meetings.

### Baseline Characteristics

Baseline demographics and PA levels are presented in Table 1. The mean age for participants was 55.4 (SD 6.0, age range 40-65) years, and most of the participants were Caucasian (n=54, 98%). Female participants made up the majority (n=51, 91%) of the sample. Most participants (n=45, 82%) had at least some college or university education, and nearly half (n=27, 49%) reported earning a gross family income of CAD $100,000 or greater. Most (n=38, 69%) participants were currently married or living with a partner. Despite all participants self-reporting accumulating less than 150 minutes of MVPA per week, the average amount of weekly MVPA accumulated was 202.7 (SD 175.4) minutes at baseline, exceeding the Canadian PA guidelines [29]. Participants on average walked 7420 (SD 3050) steps per day. Across all groups, SBP and DBP were 123.4 (SD 11.9) and 78.8 (SD 9.2) mm Hg, respectively. PPM, SFII, and the control arm all reported RAI scores of 15.7 (SD 2.4), 14.6 (SD 2.4), and 15.8 (SD 2.3), respectively.

**Table 1:** Baseline demographics.

Variable			PPM^a^ (n=19)		SFII^b^ (n=18)	CON^c^ (n=18)		*P* value	
Age (years), mean (SD)			55.4 (5.70)		55.8 (6.17)	55.1 (6.43)		.95	
**Sex, n (%)**									.47
	Male	3 (16)		1 (6)		1 (6)			
	Female	16 (84)		17 (94)		17 (94)			
**Ethnicity, n (%)**									.15
	Caucasian	19 (100)		18 (100)		17 (94)			
	South Asian	0 (0)		0 (0)		1 (6)			
**Education, n (%)**									.60
	Some high school	0 (0)		1 (6)		0 (0)			
	High school graduate	2 (11)		3 (16)		4 (22)			
	Some college or university	3 (15)		0 (0)		0 (0)			
	College or university degree	10 (53)		7 (39)		9 (50)			
	Graduate degree or higher	4 (21)		7 (39)		5 (28)			
**Yearly household income (CAD $)^d^, n (%)**									.38
	$15,000-$29,999	1 (6)		1 (6)		0 (0)			
	$30,000-$49,999	1 (6)		2 (11)		1 (6)			
	$50,000-$74,999	3 (17)		4 (22)		2 (11)			
	$75,000-$99,999	2 (11)		5 (28)		4 (22)			
	$100,000-$150,000	7 (39)		2 (11)		6 (33)			
	$150,000+	4 (22)		4 (22)		4 (22)			
**Living situation, n (%)**									.33
	Married or living with partner	12 (63)		11 (61)		15 (83)			
	Single or living alone	4 (21)		2 (11)		1 (6)			
	Single or living with others	3 (16)		5 (28)		2 (11)			
MVPA^e^ (min/week), mean (SD)			217.0 (199.3)		131.8 (157.1)	258.5 (148.8)		.09	
Daily steps, mean (SD)			7367 (3095)		7106 (3358)	7789 (2806)		.80	
SBP^f^ (mm Hg), mean (SD)			125.2 (12.0)		123.0 (10.9)	121.7 (12.5)		.80	
DBP^g^ (mm Hg), mean (SD)			80.8 (8.3)		77.5 (9.9)	77.80 (9.9)		.67	
RAI^h^ score, mean (SD)			15.7 (2.4)		14.6 (2.4)	15.75 (2.3)		.32	

^a^PPM: pay-per-minute.

^b^SFII: self-funded investment incentive.

^c^CON: control.

^d^At the time of writing: CAD $1=US $0.74.

^e^MVPA: moderate-to-vigorous physical activity.

^f^SBP: systolic blood pressure.

^g^DBP: diastolic blood pressure.

^h^RAI: relative autonomy index.

### Recruitment

With recruitment taking place over 5 weeks, the recruitment rate was 77%. Thus, the screening-to-enrollment ratio dictated that 95% of those eligible did enroll in the study, with a total of 55 participants that provided consent. Throughout the 8-week intervention, there was a 95% retention rate, with 52 of 55 randomized participants completing the study.

### App Engagement

Engagement was analyzed for the PPM and SFII arms, as the control group did not have access to the Healthy Hearts program. Lessons were presented on a completion basis, meaning you had to complete the previous lesson to unlock the next. Overall, 65% of all Healthy Hearts lessons were completed (63% for PPM and 67% for SFII).

### Intervention Acceptability: Qualitative Evaluation

A total of 52 participants (PPM: n=17; SFII: n=17; CON: n=18) completed the semistructured interview at the follow-up meeting. The main themes that emerged were positive and negative feelings about the intervention and user design of the mobile app.

The user-friendliness of the app was mentioned by most who used it (PPM n=13; SFII n=15), with positive comments relating to the usability and system interface. Ten participants mentioned that 3 lessons per week were adequate. Generally, the content was well accepted. The control group, who received weekly emails, also gave positive feedback on the variety of content received. When asked about the impact of the program on their PA, 30 participants commented on Fitbit, citing how it was a useful tool to see their daily activity.

Participants in the SFII group were asked if they would have been willing to give their own money toward their contract. A minority of participants (6/17, 32%) mentioned that they would not have been comfortable investing their own money into a PA program. Of those who said yes to investing CAD $400 into their health with a guarantee to be given the money back after 8 weeks, 86% of participants reported a gross income of greater than CAD $75,000 per year. Of those that said no to investing their own money for the duration of the program, 75% reported earning less than CAD $75,000 gross annual income.

While the user interface of the app was appreciated, some participants (n=3) did not find all the lessons necessary for them. Three different participants mentioned that they wanted more guidance with how much they were earning each week in the program, either through email or through the app.

### Preliminary Efficacy

#### Physical Activity

For MVPA at 4 weeks, both PPM and SFII showed medium effect size differences, relative to control (PPM vs control: η^2^_p_=0.06, mean 117.8, SD 514 minutes; SFII vs control: η^2^_p_=0.08, mean 145.3, SD 616 minutes). However, for MVPA, at 8 weeks SFII showed a medium effect relative to control (η^2^_p_=0.07), while small effects were observed for PPM relative to control (η^2^_p_=0.003). This translates to a mean increase in MVPA by 22.8 (SD 249) minutes per week for PPM relative to control. Meanwhile, SFII intervention showed a mean increase of 113.8 (SD 256) minutes per week relative to control. Relative to baseline, 70% (n=26) of those were meeting the Canadian PA guidelines in both financial incentive arms.

Relative to the control for daily steps, both PPM and SFII showed a small effect with changes in daily steps at both 4 (PPM η^2^_p_=0.02, mean Δdaily steps 937, SD 2039; SFII η^2^_p_≤0.001, mean Δdaily steps 274, SD 2043) and 8 weeks (PPM: η^2^_p_=0.02 mean Δdaily steps −27, SD 2362; SFII: η^2^_p_≤0.001, mean Δdaily steps −144, SD 2367) (Table 2).

**Table 2:** Changes in physical activity outcomes at 4 weeks and 8 weeks relative to baseline.

	PPM^a^ (n=19), mean (SD)		SFII^b^ (n=18), mean (SD)			CON^c^ (n=18), mean (SD)			4 weeks					8 weeks			
	Δ 4w	Δ 8w	Δ 4w	Δ 8w	Δ 4w		Δ 8w	Overall *P* value		PPM vs CON, η^2^_p_^d^	SFII vs CON, η^2^_p_	PPM vs SFII, η^2^_p_	Overall *P* value		PPM vs CON, η^2^_p_	SFII vs CON, η^2^_p_	PPM vs SFII, η^2^_p_
MVPA^e^ (minutes)	117.9 (316.0)	20.6 (201.7)	144.8 (236.7)	149.2 (214.3)	−31.6 (122.4)		−20.7 (134.2)	.08		*0.06* ^f^	*0.08*	<0.001	.15		<0.001	*0.07*	0.05
Daily steps	950 (2329)	−7 (2887)	352 (1839)	−23 (1962)	221 (2274)		183 (2906)	.54		0.02	<0.001	0.02	.83		<0.001	*0.07*	<0.001

^a^PPM: pay-per-minute.

^b^SFII: self-funded investment incentive.

^c^CON: control.

^d^η^2^_p_: partial eta squared.

^e^MVPA: moderate-to-vigorous physical activity.

^f^Italics indicate at least a medium effect in partial eta squared values.

#### Blood Pressure

Relative to the control, SBP decreased in the SFII intervention group (η^2^_p_=0.001; ΔSBP −0.4, SD 1.4 mm Hg) but increased in the PPM intervention (η^2^_p_=0.12; ΔSBP 7.1, SD 23.6 mm Hg). Similarly, relative to the control, DBP decreased in the SFII intervention group (η^2^_p_=0.02, ΔDBP −2.31, SD 7.66 mm Hg) but increased in the PPM intervention (η^2^_p_=0.04; ΔDBP 3.55, SD 6.25 mm Hg). Table 3 displays the changes in blood pressure and PA motivation.

**Table 3:** Changes in blood pressure and physical activity motivation at 8 weeks relative to baseline.

	PPM^a^ (n=19), mean (SD)	SFII^b^ (n=18), mean (SD)	CON^c^ (n=18), mean (SD)	Overall *P* value	PPM vs CON, η^2^_p_^d^	SFII vs CON, η^2^_p_	PPM vs SFII, η^2^_p_
	Δ8w	Δ8w	Δ8w				
SBP^e^ (mm Hg)	1.4 (5.5)	−5.5 (8.5)	−4.8 (12.3)	.08	*0.12* ^f^	<0.001	*0.13*
DBP^g^ (mm Hg)	0.2 (4.9)	−4.0 (6.9)	−1.9 (11.8)	.17	0.04	0.02	*0.12*
RAI^h^	−2.8 (3.6)	−3.9 (2.5)	−3.3 (3.3)	.27	<0.001	0.03	0.05

^a^PPM: pay-per-minute.

^b^SFII: self-funded investment incentive.

^c^CON: control.

^d^η^2^_p_: partial eta squared.

^e^SBP: systolic blood pressure.

^f^Italics indicate at least a medium effect in partial eta squared values.

^g^DBP: diastolic blood pressure.

^h^RAI: relative autonomy index.

#### Relative Autonomy Index

At the 8-week follow-up, the PPM arm decreased their score by 0.3 (SD 1.4; η^2^_p_=0.01) relative to the control, and the SFII arm decreased their score by 1.3 (SD 4.3; η^2^_p_=0.03) relative to the control. These reductions translate to a small effect size.

## Discussion

### Overview

The primary objective of this study was to determine the feasibility (recruitment, engagement, and acceptability) of an 8-week mobile-based PPM and SFII hypertension prevention program. The secondary objectives of this study were to explore the effects of PPM and SFII interventions relative to the control on improving PA levels, BP, and PA motivation following the intervention. To our knowledge, this is the first mobile app intervention to compare the PPM and SFII financial incentive arms, relative to a control group. Overall, the findings from this study support a future efficacy trial in line with Phase III of the ORBIT model [23]. The modified SFII intervention evaluated in this study may be a sustainable financial incentive to promote PA. Future studies with larger sample sizes and longer study periods are warranted.

### Principal Findings

Based on previous research [21,49], it was hypothesized that recruitment for this study would be feasible at >70%. Ryde et al [49] analyzed the characteristics for success in 30 workplace PA interventions and categorized a recruitment rate ≥70% as high. While the settings and durations of these interventions varied, it was assumed that the employee population included adults, and thus is comparable to this sample. Compernolle et al [21] had a recruitment rate of 83% for the mHealth study and recruited 28 older adults for a 3-week intervention. While these comparator studies did not target adults specifically aged 40-65 years at risk for hypertension, they did use PA-improving strategies or were offered through mHealth technologies. In this study, the recruitment was 77% over 5 weeks, which was comparable to these previous studies [21,49].

Throughout the 8-week intervention, there was a 95% retention rate, with 52 of 55 randomized participants completing the study. An 80-100% retention rate is indicative of a strong trial [50]. The retention is much higher compared to previous web-based and mHealth interventions (50%-80%) that have reported high dropout rates [51,52]. However, the current retention rate is comparable to other digital behavior change interventions (90%-95%) [21]. Retention rates among financial incentive studies typically increase with the value of the monetary incentive offered [53].

Previous research has shown that maintaining engagement over time is a challenge in many mHealth interventions [54]. Low user engagement typically leads to poor intervention effectiveness and adherence [55]. Of those in the incentive arms that received the Healthy Hearts program, user engagement was acceptable with 65% completing the program and with 75% using the app 4 weeks in. Engagement usage metrics vary among studies, and thus finding a similar study was a challenge. However, in an RCT of 125 parent-child dyads, it was reported that 53.5% (SD 37.6%) of mHealth content relating to family weight loss that was delivered in the 16-week intervention was accessed [22]. Thus, we considered a 65% competition rate in this feasibility study a success. Future studies need to explore other engagement methods explored in digital PA studies, which include the number of app logins and duration of use [56,57], days and minutes of use [58], and monitoring use of the app (ie, logging in a PA diary) [56,59].

Overall, positive feedback was received on the program. Both objective usage metrics and subjective experiences with the Healthy Hearts program delivered using the Pathverse platform showed that adults were highly engaged with this intervention. Many commented that completing 3 lessons per week was an adequate amount that did not overwhelm them. These findings are all indicators of the acceptability of the intervention to this demographic.

The study results supported the hypothesis that those in the PPM or SFII arms, relative to control, would show a small-to-moderate effect size in improving MVPA and daily steps at 4 and 8 weeks, respectively. Small-to-moderate effect sizes have been documented for overall increases in PA in previous financial incentive and PA studies [15,60]. From these findings, we recommend that a sample of at least 306 participants, with 102 people per arm, would ensure 80% power to detect a 0.18 difference between the intervention arms and the control arm.

Although we did not ask participants to commit to investing their own money in the SFII group, over 68% of the participants in the study said that they are willing to invest their own money. Participants found the 5% return from SFII acceptable. This is important to the feasibility of the investment-based SFII model proposed in this study since several stock index funds over the last 30 years have shown an average return between 5% and 8% [30]. Thus, it may be possible for this SFII model to be employed by insurance, government, or private firms, where employees may have the option to reinvest a portion of their paycheck if they are meeting a certain behavior outcome.

Contrary to our hypothesis, participants in the PPM and SFII groups did not show a small-to-moderate reduction in SBP and DBP, relative to the control. Previous studies of similar length have reported a significant reduction in SBP and DBP by −3.8 mm Hg (95% CI−5.63 to−2.06 mm Hg; *P<*.01) and −2.1 mm Hg (95% CI−3.51 to −0.65 mm Hg; *P<*.05), respectively [61]. The small effect size observed in this study may be due to a floor effect as the mean baseline BP was 123/79 mm Hg. We recommend a research assistant to perform BP measurements for a stage III RCT.

Finally, it was hypothesized that those in the PPM and SFII arm would increase their autonomous motivation due to receiving an 8-week hypertension education program and receiving a modest incentive. However, the results of this study did not align with our hypothesis. The design of this program encouraged competence development in promoting reaching attainable PA goals, a strategy that has the potential to increase intrinsic motivation through the self-determination theory [62]. Previous studies have shown that extrinsic rewards may be used to fulfill these psychological needs to avoid harming intrinsic motivation by rewarding achievements of realistic self-regulatory goals (eg, monitoring MVPA), and providing choices to the participants for the types of reward and the activities [63]. It may be possible that a longer intervention duration may be required to improve intrinsic motivation [64].

### Limitations

There were several study limitations. First, the participants were primarily Caucasian, with higher education, and with most earning above CAD $100,000 annually, well above the median income for British Columbians [65], and therefore do not represent the general population. Recruiting through Facebook may have presented this recruitment bias [66], thus limiting this study’s generalizability beyond those with internet access and a Facebook account. Second, the use of self-report for PA levels at baseline also introduced a reporting bias. Third, since this study was completed virtually, it lacked the consistency of having a trained research assistant measure participants’ BP. Over half (56%) of participants had access to a personal BP cuff at baseline, but due to pharmacies removing BP cuffs during to the COVID-19 pandemic and current physical distancing measures in effect [67], in-person laboratory BP measurements were not possible.

### Conclusions

This study examined the feasibility of an 8-week SFII and PPM financial incentive mHealth intervention to improve PA and collected both quantitative and qualitative data. Feasibility results indicated high recruitment and retention rates, engagement, and acceptability. Preliminary results showed PPM and SFII showed a small-to-medium effect in improving MVPA and steps relative to the control. SFII may have the potential to be more sustainable than a PPM financial incentive model due to the nature of the self-funding incentive. It is recommended that this framework of financial incentive be explored in practice with participants investing their own money for the duration of the intervention, opposed to a mock contract agreement. Overall, the results from this study support recommendations for a future full-scale RCT in line with Phase III of the ORBIT model [23].

## Appendix

Multimedia Appendix 1 CONSORT-eHEALTH checklist (V 1.6.1).

## Abbreviations

BP: blood pressure
BREQ-3: Behavioral Regulation in Exercise Questionnaire
DBP: diastolic blood pressure
mHealth: mobile health
M-PAC: multiprocess action control
MVPA: moderate-to-vigorous physical activity
PA: physical activity
PPM: pay-per-minute
RAI: relative autonomy index
RCT: randomized controlled trial
SBP: systolic blood pressure
SFII: self-funded investment incentive

## Data Availability

The data sets generated and analyzed during the current study are available from the corresponding author on reasonable request.
